# Abnormal Voxel-Wise Degree Centrality in Patients With Late-Life Depression: A Resting-State Functional Magnetic Resonance Imaging Study

**DOI:** 10.3389/fpsyt.2019.01024

**Published:** 2020-01-31

**Authors:** Jun Li, Hengfen Gong, Hongmin Xu, Qiong Ding, Naying He, Ying Huang, Ying Jin, Chencheng Zhang, Valerie Voon, Bomin Sun, Fuhua Yan, Shikun Zhan

**Affiliations:** ^1^ Department of Functional Neurosurgery, Ruijin Hospital, Shanghai Jiao Tong University School of Medicine, Shanghai, China; ^2^ Department of Psychiatry, Shanghai Pudong New Area Mental Health Center, Tongji University School of Medicine, Shanghai, China; ^3^ Department of Radiology, Ruijin Hospital, Shanghai Jiao Tong University School of Medicine, Shanghai, China; ^4^ Neural and Intelligence Engineering Center, Institute of Science and Technology for Brain-Inspired Intelligence, Fudan University, Shanghai, China; ^5^ Department of Psychiatry, University of Cambridge, Cambridge, United Kingdom

**Keywords:** late-life depression, resting state, functional magnetic resonance imaging, degree centrality, onset age

## Abstract

**Objectives:**

Late-life depression (LLD) has negative impacts on somatic, emotional and cognitive domains of the lives of patients. Elucidating the abnormality in the brain networks of LLD patients could help to strengthen the understanding of LLD pathophysiology, however, the studies exploring the spontaneous brain activity in LLD during the resting state remain limited. This study aimed at identifying the voxel-level whole-brain functional connectivity changes in LLD patients.

**Methods:**

Fifty patients with late-life depression (LLD) and 33 healthy controls were recruited. All participants underwent a resting-state functional magnetic resonance imaging scan to assess the voxel-wise degree centrality (DC) changes in the patients. Furthermore, DC was compared between two patient subgroups, the late-onset depression (LOD) and the early-onset depression (EOD).

**Results:**

Compared with the healthy controls, LLD patients showed increased DC in the inferior parietal lobule, parahippocampal gyrus, brainstem and cerebellum (*p <* 0.05, AlphaSim-corrected). LLD patients also showed decreased DC in the somatosensory and motor cortices and cerebellum (*p* < 0.05, AlphaSim-corrected). Compared with EOD patients, LOD patients showed increased centrality in the superior and middle temporal gyrus and decreased centrality in the occipital region (*p <* 0.05, AlphaSim-corrected). No significant correlation was found between the DC value and the symptom severity or disease duration in the patients after the correction for multiple comparisons.

**Conclusions:**

These findings indicate that the intrinsic abnormality of network centrality exists in a wide range of brain areas in LLD patients. LOD patients differ with EOD patients in cortical network centrality. Our study might help to strengthen the understanding of the pathophysiology of LLD and the potential neural substrates underlie related emotional and cognitive impairments observed in the patients.

## Introduction

Depression refers to a mental disorder characterized by low mood present across most situations for at least two weeks. Late-life depression (LLD) can be defined as a major depressive episode occurring in old age, usually over 60 years of age. Aside from the emotional and somatic burdens associated with depression, such as insomnia, anorexia and fatigue (1), elderly depressive patients may also show impairment in various cognitive domains including attention (2), memory (3, 4), information processing speed (5, 6), and executive functions (4, 7). All these somatic, emotional, and cognitive abnormalities may severely affect the life quality of the patients.

Elucidating the brain abnormality of LLD patients could help to strengthen the understanding of LLD pathophysiology, and develop effective interventions. Grey matter alterations have been reported in multiple brain areas including the frontal, parietal regions and limbic system in LLD patients (8, 9). Several task-based functional magnetic resonance imaging (fMRI) studies have also indicated abnormal functional activities in the areas of frontal lobes and limbic system (10, 11). While structural alterations reflect the long-term influence of depression, and task-based imaging profiles the altered brain reaction to external stimuli or under specific situations, resting-state fMRI explores the intrinsic changes of brain activity in the state without any influence of external stimuli. Resting-state fMRI studies using the method of independent component analysis has revealed that the default mode network, the frontoparietal network and the sensorimotor network showed abnormal connectivity in LLD patients (12, 13). LLD patients also exhibited abnormal local synchronization in various brain areas, as revealed by the measure of regional homogeneity (14). These observations suggest that the brain activities in the resting state have extensive cortical and subcortical abnormalities in LLD patients, which might be associated with clinical manifestations such as emotional disturbance and cognitive deficits observed in the patients.

Degree centrality (DC), an index of the total weights of connections for a given node, has recently been applied to reveal the core hub architecture of brain networks (15). Increased voxel-wise DC in a brain region indicates an elevated degree of its global connectivity, and decreased voxel-wise DC in a brain region suggests a reduced degree of its global connectivity. Voxel-wise DC has been applied to reveal the abnormal brain networks in various types of neurological or psychiatric diseases (16–22). This method has also been used to compare the brain network features of different psychiatric diseases with potentially similar neural pathology, such as autism and attention-deficit hyperactivity disorder (23), or to examine the brain-network difference between the subtypes of a disease such as the Parkinson’s disease patients with depression and those without depression (24). The alterations of the whole-brain degree centrality in young patients with major depression have been illustrated in two recent studies revealing that the frontoparietal network, the limbic system, and the striatal areas show DC abnormality in the patients (25). However, depression-specific alterations of network centrality among LLD patients yet remain to be identified.

LLD can be divided into the early-onset depression (EOD, depression occurred for the first time under the age of 60) and late-onset depression (LOD, depression occurred for the first time over the age of 60) according to the onset age of depression (most studies use 60 years as the onset age to distinguish EOD and LOD). While some studies indicate that the LOD patients are not different from the EOD patients in most aspects such as etiological factors, phenomenology or clinical outcomes (26), other studies suggest that the LOD patients show a more severe emotional burden and more extensive cognitive impairments than EOD patients (27–29). Several imaging studies have indicated differences in structure or task-based functional activity between EOD and LOD patients in a wide range of brain areas, including the medial and lateral frontal areas, hippocampus and amygdala (30–35). However, only a few studies have examined the difference in resting-state functional network between EOD and LOD patients. One study using regional homogeneity and the other study using amplitude of low-frequency fluctuation as the indices suggest the local synchronization and low-frequency fluctuation differ in multiple cortical areas and brainstem between LOD and EOD patients (36, 37). The centrality profile of the resting-state brain networks has not been elucidated between LOD and EOD patients.

In the present study, we hypothesized that the architecture of brain networks reflected by degree centrality may show abnormality in cortical and subcortical areas in the LLD patients.

To explore the alterations of functional centrality in LLD patients, we examined the difference in voxel-wise DC between the LLD patients and healthy controls (HCs). To further examine the hypothesis that a difference of the architecture of brain networks may exist between EOD and LOD patients, we compared the DC between the two subgroups of patients. The relationship between the centrality indices of the brain areas being identified abnormal and the clinical assessment was also examined.

## Methods

All procedures used in the present study were approved by the Ethics Committee of Shanghai Pudong New Area Mental Health Center (approval letter: PDJWLL2014001). Patients with LLD and HCs were recruited *via* advertisements and were fully instructed regarding experimental procedures. All participants gave their written informed consent in accordance with the Declaration of Helsinki.

### Participants

Patients diagnosed with major depression according to the criteria in the International Classification of Diseases (ICD-10) by a physician were recruited at Pudong New Area Mental Health Center in Shanghai, China. Healthy volunteers whose age matched to ±10 years with the patients were recruited as controls. Participants aged under 60 or over 80, with a history of severe head trauma, with alcohol abuse, with psychiatric diseases other than depression, with claustrophobia, or with metal or electronic implants were excluded. Patients were using tricyclic, selective serotonin reuptake inhibitors or serotonin and noradrenaline reuptake inhibitors when they were recruited.

### Demographics and Clinical Assessment

Basic demographics (i.e. gender, age, education level, handedness) of the participants were collected. Both the patients and HCs filled out the Hamilton Depression Scale (HAMD) (38) right before the resting-state fMRI scan. Patients were asked to report the onset age and the duration of depression. In addition, Mini-Mental State Examination (MMSE) [Folstein, (39)] was carried out and potential participants scored lower than 21, who may have moderate to severe cognitive impairment, were excluded from the study. Mann–Whitney test was used for the between-group comparisons of age, disease duration, and scores of HAMD and MMSE. Chi-square test was used for the between-group comparisons of gender and education level.

### Image Acquisition

Resting-state fMRI was performed on a GE Signa HDxt 3.0 T MRI scanner using an eight-channel phased-array head coil. Each participant lay supine with their head snugly fixed by foam pads. The participant was asked to keep still as long as possible and to keep his/her eyes closed but remain awake. Resting-state fMRI was obtained using an echo-planar imaging sequence with protocols of TR = 2000 ms, TE = 30 ms, flip angle = 90°, FOV 240 mm × 240 mm, matrix = 64 × 64, voxel size 3.75 mm × 3.75 mm × 4.00 mm, 35, 37 or 39 axial slices, 210 volumes acquired in 7 min.

### Imaging Data Preprocessing

Preprocessing of resting-state fMRI data was conducted using Data Processing Assistant for Resting-State fMRI (DPARSF; http://rfmri.org/dparsf) software (version 4.5) embedded in Data Processing and Analysis for Brain Imaging (DPABI; http://rfmri.org/dpabi) toolbox (version 4.1) on the MATLAB platform (MathWorks, Natick, MA, USA). In brief, the first 10 volumes of each functional time series were discarded, as the participants were adjusting themselves to the fMRI environment during that period. The remaining 200 images were slice-time-corrected with the 35th, 37th or 39th slice as the reference and spatially realigned for head motion. Head motion was assessed by evaluating three translations and three rotations for each scan. Translational thresholds were set to ± 3 mm, while rotational thresholds were limited to ± 3°. After head motion correction, functional images were spatially normalized to Montreal Neurological Institute (MNI) space using echo-planar imaging sequence templates (resampled voxel size 3 mm × 3 mm × 3 mm). All images were linearly detrended and bandpass-filtered (0.01–0.1 Hz) to have the high-frequency respiratory and cardiac noises reduced. The white matter signal, cerebrospinal fluid signal, and Friston 24 head motion parameters were regressed out from the time courses of every voxel.

### Voxel-Wise Degree Centrality

The value of degree centrality was calculated using DPARSF (http://rfmri.org/dparsf). For each voxel, the blood-oxygen-level-dependent (BOLD) time course was extracted and the Pearson correlation coefficients with every other voxel in the brain were calculated. A matrix of Pearson correlation coefficients between any pair of voxels was obtained to construct the whole-brain functional connectivity matrix for each participant. Finally, the resulting matrices (DC maps) were smoothed with a Gaussian kernel (full-width half maximum = 6 mm) to enable group comparisons.

To obtain the spatial distribution of DC maps for the HC group and the LLD group, the averaged DC map was calculated for each group using the image calculator module embedded in DPABI. The resulting averaged DC maps were overlaid on rendering views with BrainNet Viewer (http://www.nitrc.org/projects/bnv).

To determine the abnormality in core brain hub architecture as reflected by DC in the LLD patient group, we identified the clusters with DC difference between the LLD patients and HCs, using two-sample *t*-test with gender, age, education level, score of MMSE and framewise displacement regressed out. To determine the difference of DC between LOD patients and EOD patients, another two-sample *t*-test was performed between the LOD group and EOD group in the same manner. AlphaSim correction was used for multiple comparisons to achieve a corrected *p <* 0.05 determined by the Monte Carlo simulation by a combination of a voxel-wise threshold of *p <* 0.001 and a minimum cluster size calculated by the AlphaSim program embedded in DPABI (https://afni.nimh.nih.gov/pub/dist/doc/manual/AlphaSim.pdf), The resulting *t* maps were overlaid on rendering views with BrainNet Viewer and on axial views in slices with the viewer module embedded in DPABI (http://rfmri.org/dpabi), and the anatomy of surviving brain regions was reported using xjView software (http://www.alivelearn.net/xjview).

To further examine whether there exists a relationship between the abnormal DC values identified in the patients and the related clinical assessment (onset age of depression, duration of depression and HAMD score), Pearson's correlation was respectively calculated in the LLD group and two subgroups. The averaged DC values of abnormal brain regions for the relationship analyses were extracted using spheres of 6-mm radius centering at the coordinates with peak statistical difference from the between-group comparisons (LLD group vs. HC group, or LOD group vs. EOD group) using the signal extractor module embedded in DPABI. To control for multiple comparisons, we used a Bonferroni correction of *p* = 0.016 as the significant statistical threshold (three correlations were examined for each spherical region of interest).

## Results

### Demographics and Clinical Assessment of Participants

The detailed demographics and results of the clinical assessment of the participants are illustrated in **Table 1**. The 50 LLD patients (19 males, 31 females) had a mean age of 66.6 ± 0.7 (range: 60–78) years, 84.0 ± 17.2 months of disease duration, mean scores of 21.9 ± 1.5 on HAMD and 28.1 ± 0.3 on MMSE. The 33 HCs (17 males, 16 females) had a mean age of 67.2 ± 0.8 (range: 60–78) years. All participants were right-handed. The gender, age and mean score on MMSE were not significant between the patients and healthy controls (*p*s *>* 0.091). The education level (*p* = 0.043) and score on HAMD (*p <* 0.001) differed significantly between the groups.

**Table 1:** Demographics and clinical assessment of the participants.

	LLD Patients		HC	*p* value
**Numbers**	50		33	
**Gender (M/F)**	19/31		17/16	0.224
**Age (year)**	66.6 ± 0.7		67.2 ± 0.8	0.499
**Education (I/E/S/H/C)**	1/5/32/6/6		1/4/10/8/10	0.043*
**Onset age (year)**	59.6 ± 1.6		NA	
**Duration (month)**	84.0 ± 17.2		NA	
**HAMD-24**	21.9 ± 1.5		2.4 ± 0.6	<0.001*
**MMSE**	28.1 ± 0.3		28.9 ± 0.2	0.091
****	**LOD patients**	**EOD patients**	***p* value**	
**Numbers**	29	21		
**Gender (M/F)**	14/15	5/16	0.079	
**Age (year)**	68.5 ± 1.0	64.0 ± 0.7	0.002*	
**Education (I/E/S/H/C)**	1/2/16/5/5	0/3/16/1/1	0.249	
**Onset age (year)**	66.7 ± 1.0	49.9 ± 2.3	<0.001*	
**Duration (month)**	22.4 ± 4.8	169.1 ± 32.5	<0.001*	
**HAMD-24**	21.7 ± 2.1	22.3 ± 2.3	0.911	
**MMSE**	28.2 ± 0.4	28.0 ± 0.5	0.980	

C, college and above; E, elementary school; EOD, early-onset depression; F, female; HAMD, H, high school; Hamilton Depression Scale; HC, healthy control; I, illiteracy; LOD, late-onset depression; M, male; MMSE, Mini-Mental State Examination; p, probability; S, secondary school. * indicates significant difference (p < 0.05) between the groups.

The demographics and clinical assessment of EOD patients and LOD patients are also illustrated in **Table 1**. The two subgroups of patients did not differ in gender, education, scores on HAMD or MMSE (*p*s *>* 0.079). There was a significant between-group difference of age (LOD: 68.5 ± 1.0, EOD: 64.0 ± 0.7, *p* = 0.002). Gender, age, education level and score on MMSE were used as covariates in the between-group comparisons of DC maps, in order to exclude the potential impact from demographics.

### DC Difference Between LLD Patients and HCs

**Figure 1:**
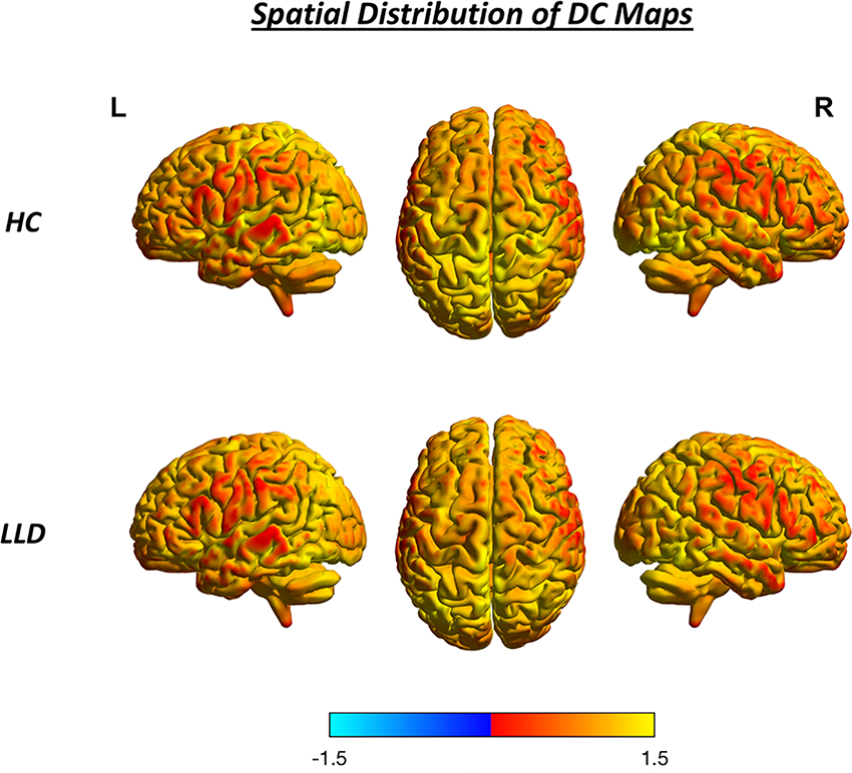
Spatial distribution of DC maps in the HC group and the LLD patient group. Color bars indicate DC values; brighter color indicates DC values higher than the whole-brain average. HC, healthy control; L, left; LLD, late-life depression; R, right.

**Figure 2:**
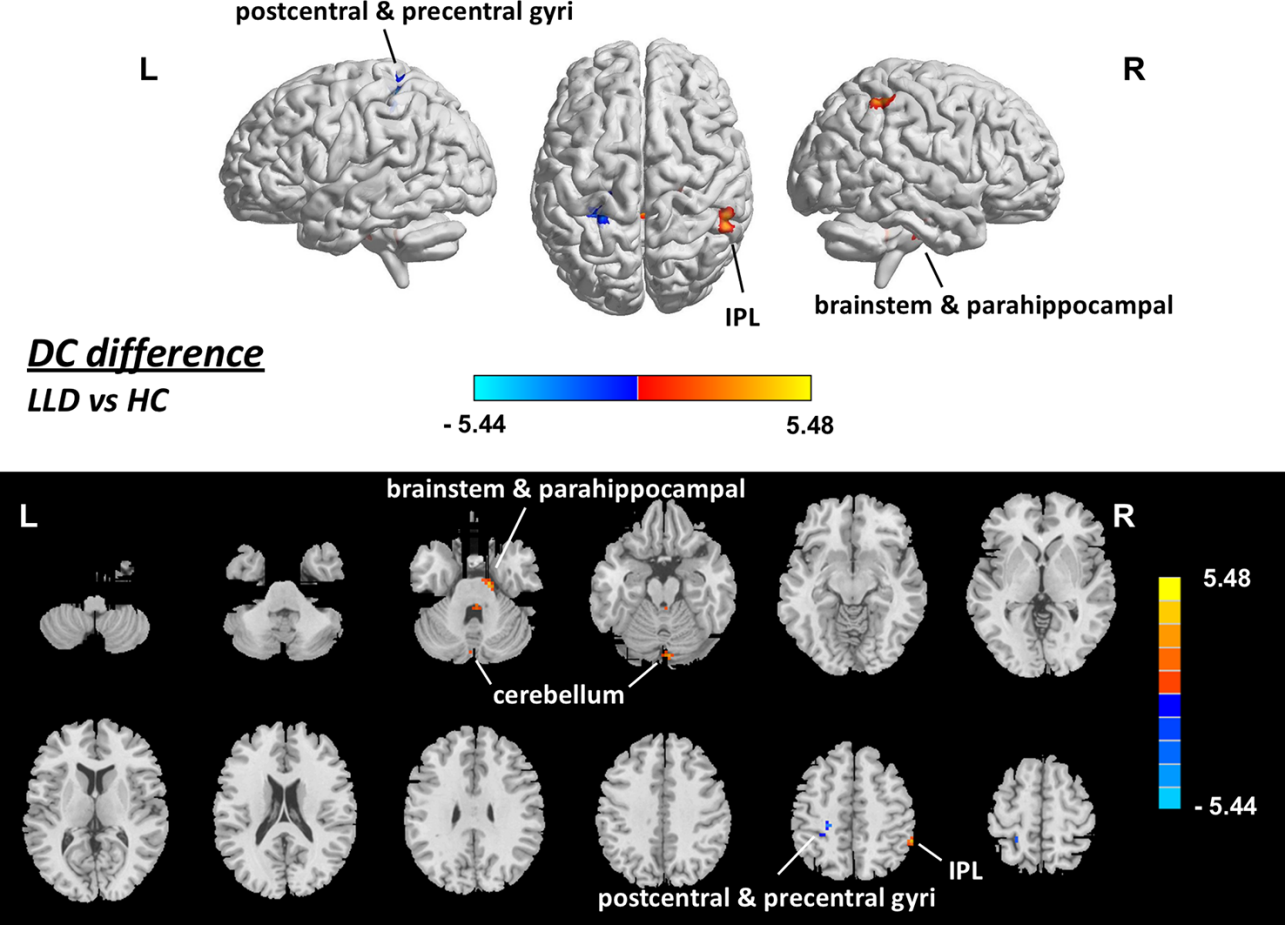
Brain areas showing difference in DC between LLD patients and HCs (*p < *0.05, corrected), with gender, age, education, score on MMSE and framewise displacement regressed out. Upper: rendering views. Lower: axial slice views. Color bars indicate *T*-score; a warm color indicates areas where DC value in LLD patients > DC value in HCs, a cold color indicates areas where DC value in LLD patients < DC value in HCs. DC, degree centrality; HC, healthy control; IPL, inferior parietal lobule; L, left; LLD, late-life depression; R, right.

The mean framewise displacement of the LLD group (0.20 ± 0.02 mm) was slightly larger than that of the HC group (0.14 ± 0.01 mm, *p =* 0.009), and thus was also used as a covariate in the statistical analyses of DC. DC maps for the HC group and the LLD group are presented in **Figure 1**. The estimated smoothness of the DC map is FWHMx = 6.203 mm, FWHMy = 6.402 mm, FWHMz = 6.169 mm, dLh = 0.512. AlphaSim correction is performed with a combination of voxel *p <* 0.001 and cluster size > 12. Compared with the HCs, the LLD patients showed increased DC in the right inferior parietal lobule, right parahippocampal gyrus, right cerebellum and bilateral brainstem (*p <*0.05, AlphaSim-corrected, **Figure 2**, **Table 2**). The LLD patients showed decreased DC in the brain areas of the left precentral and postcentral gyri, and the left cerebellum (*p <* 0.05, AlphaSim-corrected, **Figure 2**, **Table 2**).

**Table 2:** Brain areas showing different DC between LLD patients and HCs (*p <* 0.05, corrected).

Region (AAL name)	Peak MNI coordinate			Voxel size	Peak *T* value
	*x*	*y*	*z*		
**LLD > HC**					
Parietal_Inf_R	54	−45	54	13	4.94
Parahippocampal Gyrus_R	15	−15	−27	29	5.21
Brainstem_R/L	3	−39	−33	19	4.42
Cerebelum_Crus2_L	−3	−84	−27	19	4.71
**LLD < HC**					
Postcentral_L	−21	−42	57	17	−4.27
Precentral_L	−24	−30	54	13	−5.44
Cerebelum_8_L	−24	−69	−57	13	−4.22

AAL, automated anatomical labeling; HC, healthy control; L, left; LLD, late-life depression; MNI, Montreal Neurological Institute; R, right.

The result with AlphaSim correction by a combination of voxel *p <* 0.01 and cluster size >29 is also presented in the supplementary material (**Figure S1**, **Table S1**) for reference only, in case some brain areas with potential differences couldn't survive the voxel p level of 0.001 due to the relatively small sample size.

### DC Difference Between LOD Patients and EOD Patients

**Figure 3:**
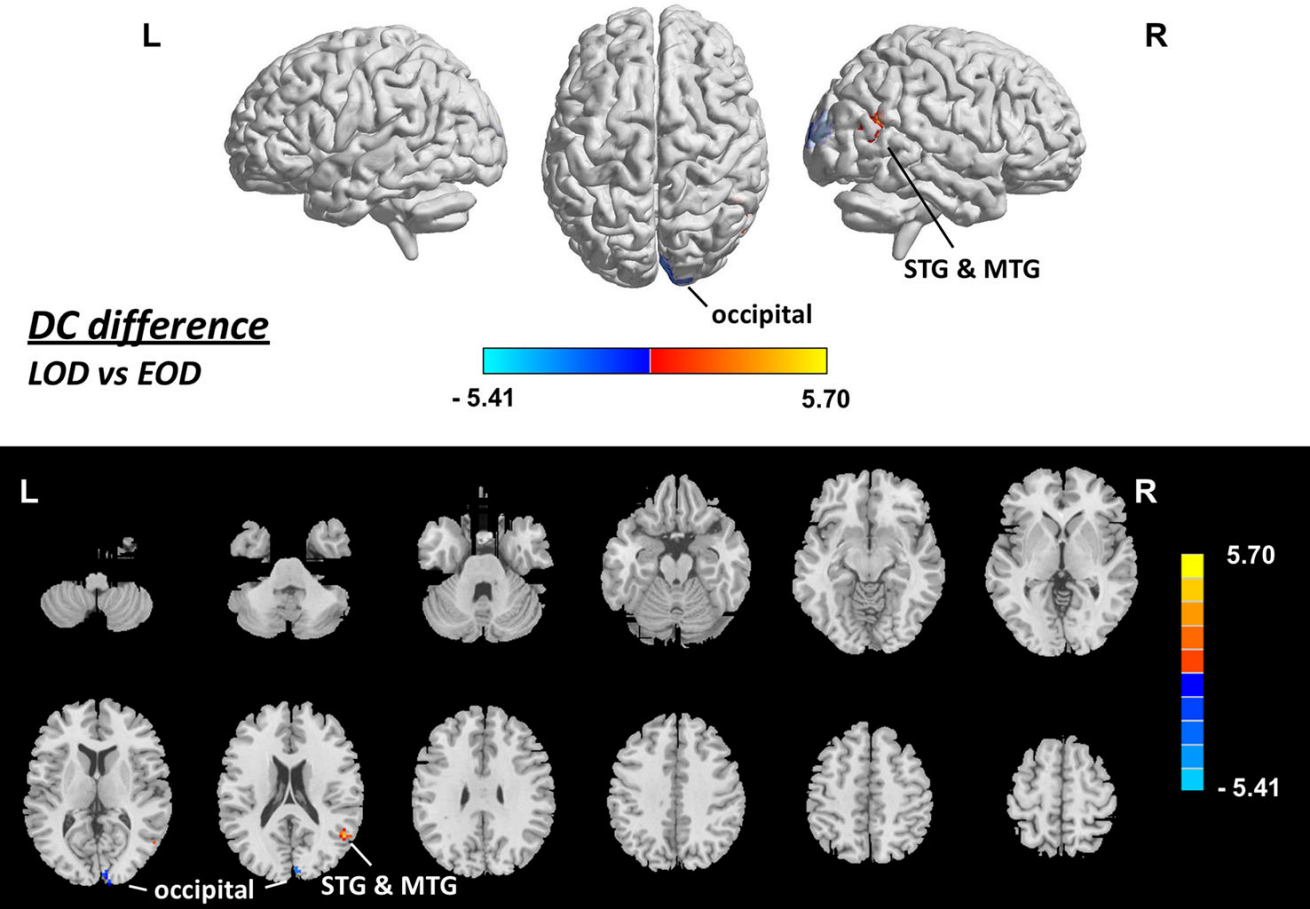
Brain areas showing difference in DC between LOD and EOD patients (*p < *0.05, corrected), with gender, age, education, score on MMSE and framewise displacement regressed out. Upper: rendering views. Lower: axial slice views. Color bars indicate *T*-score; warm color indicates areas whose DC value in LOD patients > DC value in EOD patients, cold color indicates areas whose DC value in LOD patients < DC value in EOD patients. EOD, early-onset depression; HC, healthy control; L, left; LOD, late-onset depression; MTG, middle temporal gyrus; R, right; STG, superior temporal gyrus.

Brain areas with a statistical difference in DC between the LOD patients and EOD patients were examined using between-group comparison. The mean framewise displacement of the LOD patient group (0.14 ± 0.02 mm) was not different from that of the EOD patient group (0.15 ± 0.02 mm, *p =* 0.158). The estimated smoothness of the DC map is FWHMx = 6.123 mm, FWHMy = 6.306 mm, FWHMz = 6.065 mm, dLh = 0.539. AlphaSim correction is performed with a combination of voxel *p <* 0.001 and cluster size >12. Compared with the EOD patient group, the LOD patient group showed increased DC in the area of right superior and middle temporal gyri (*p <* 0.05, AlphaSim-corrected, **Figure 3**, **Table 3**). Decreased DC was found in the right cuneus in the LOD patients, compared with the EOD patients (*p <* 0.05, AlphaSim-corrected, **Figure 3**, **Table 3**).

**Table 3:** Brain areas showing different DC between LOD and EOD patients (*p* < 0.05, corrected).

Region (AAL name)	Peak MNI coordinate			Voxel size	Peak *T* value
	*x*	*y*	*z*		
**LOD > EOD**					
Temporal_Sup_R	51	−57	21	13	5.27
Temporal_Mid_R	54	−63	12	13	5.70
**LOD < EOD**					
Cuneus_R	12	−93	12	36	−5.41

AAL, automated anatomical labeling; EOD, early-onset depression; L, left; LOD, late-onset depression; MNI, Montreal Neurological Institute; R, right.

The result with AlphaSim correction by a combination of voxel *p <* 0.01 and cluster size >28 is also presented in the supplementary material (**Figure S2**, **Table S2**) for reference only.

### Relationship of DC With Clinical Assessment

The relationship of DC with clinical assessment was explored by the calculation of Pearson's correlation. No significant correlation was found between the DC and the clinical assessment values (onset age of depression, disease duration or HAMD score) after the Bonferroni correction for multiple comparisons in the LLD patient group (*p*s *>* 0.094), or in the two subgroups (LOD: *p*s *>* 0.056; EOD group: *p*s *>* 0.094).

## Discussion

In the present study, the voxel-wise whole-brain functional connectivity in patients with late-life depression was explored using resting-state fMRI techniques. We found a unique pattern of alterations in the brain activity of the patients. Centrality indices, measured by voxel-wise degree centrality, were found to be abnormal in late-life depressive patients in the somatosensory-motor areas, inferior parietal lobule, parahippocampal gyrus, cerebellum, and brainstem. Furthermore, differentiated cortical areas with DC values were observed in the LOD patients compared with the EOD patients, in the posterior temporal gyrus and occipital region. No correlation was found between the abnormal centrality indices and the clinical assessment in the patients. The general function of brain areas with an abnormal degree centrality in the LLD patients, as well as the difference between EOD and LOD subgroups, may strengthen the understanding of the intrinsic neural-network profiles of LLD.

The somatosensory cortex receives all sensory inputs from the body, and it is responsible for somatosensory perception (40). Abnormal somatic symptoms such as somatization, defined as physical symptoms developed as a result of stress or emotional problems, have often been observed in depressive patients (41, 42). Alteration of activity in the somatosensory cortex has been suggested to be involved in the neural underpinnings of somatic symptom disorder (43). The motor cortex and cerebellum are regarded as important brain regions related to voluntary movement and motor control (44, 45). Deficits in motor-related functions have recently been observed in LLD patients (46). Thus, our observation of decreased DC in somatosensory-motor cortices and altered cerebellar DC in the LLD patients might be related to the somatosensory abnormalities and motor deficits associated with depression.

The inferior parietal lobule, a major network hub of the human brain, is involved in a broad range of behaviors and functions from bottom-up perception to social cognition and plays as important nodes in multiple network, including the frontoparietal control network, default mode network, cingulo-opercular network and ventral attention network (47). A resting-state fMRI study indicated that the connectivity between the dorsomedial prefrontal cortex and the inferior parietal lobule is related with negative self-focused thought associated with depressive symptoms in the patients with major depression (48). Our observation of increased DC at inferior parietal lobule indicates an intrinsic functional alteration in this parietal hub area in the LLD patients.

The parahippocampal gyrus is a limbic structure mainly associated with visuospatial processing and episodic memory (49). Previous studies have revealed that LLD is associated with impaired visuospatial memory and episodic memory (50, 51). The white matter integrity of parahippocampal gyrus was found disrupted in the LLD patients (52). An fMRI study indicated abnormal activation of parahippocampal gyrus while performing a memory task in LLD patients (53). Another study including 1,017 participants from the Human Connectome Project revealed that increased functional connectivity of the parahippocampal gyrus is associated with poor sleep quality and depressive problems scores (54). Thus, the increased centrality in parahippocampal gyrus observed in the present study may be related with impaired memory and depressive symptoms and poor sleep quality in the LLD patients.

The brainstem is an important structure that regulates autonomic functions, relays sensory and motor information, and modulates cognition, mood, and emotions. The brainstem is particularly critical in the modulation of emotion, as it is the home to a group of modulatory neurotransmitters such as serotonin, dopamine, and norepinephrine (55). Imaging studies have reported structural and functional abnormalities in the brainstem of patients with major depression (56, 57). Some studies also indicate that the brainstem aminergic nuclei is closely associated with late-life depressive symptoms (58, 59). Our observation of decreased network centrality of brainstem confirms an intrinsic functional abnormality of brainstem nuclei in the late-life depressive individuals.

Besides the brain areas with abnormal DC between LLD patients and HCs, we also observed DC differences between LOD patients and EOD patients, which supports the perspective that the LOD patients and EOD patients have differentiated intrinsic brain networks. The posterior middle temporal gyrus has been suggested to play critical roles in the integration of automatic information retrieval and executively-demanding goal-oriented cognition (60). The occipital cortex is mainly responsible for visual stimulus processing (61). Some studies have revealed that LOD patients have more extensive deficits than EOD patients in some cognitive domains, including the realm of memory and visual-spatial processing (27–29). In the present study, brain areas of posterior temporal gyrus and occipital areas were found with different DC values in the LOD patients and the EOD patients. It is tempting to assume that the differentiated levels of DC may be associated with the neural basis for different degrees of impairment in high cognition between LOD and EOD patients.

The study had several limitations. First, the negative result of the relationship of DC with clinical assessment (depression severity, duration of depression, and onset age) indicates that the abnormality in DC might not directly contributed to depression-related manifestations, and suggests that the abnormality might be associated with the somatic and emotional burden, and cognitive deficits other than depression itself. However, the resting-state fMRI was performed without tasks measuring emotional and cognitive processing in the patients. In future studies, the relationship of abnormal centrality indices and the emotional processing/cognitive functions needs to be examined by introducing tasks being tested in the patients. Second, although the abnormality of DC in LLD patients has been identified, the sample size of the present study is relatively small. The negative effects of DC difference in the prefrontal regions and the negative result of the relationship between DC and clinical assessment indicates may be attributed to the relatively small sample size. The findings need to be confirmed in future studies using large sample sizes. Third, the antidepressants the patients took may affect the resting-state brain activity. Our findings also did not assess the role of medicine elution which may contribute to the abnormality of the brain-network profile of the patients.

## Conclusion

Our findings in the present study indicate that the voxel-wise DC displays abnormality in LLD patients in a wide range of brain areas, which might be associated with the sensorimotor- and emotion-related alterations, and cognitive impairments observed in the patients. Also, there exists a difference in DC patterns between EOD and LOD patients. Our study might help to strengthen the understanding of the pathophysiology of LLD.

## Data Availability Statement

The datasets generated for this study are available on request to the corresponding authors.

## Ethics Statement

The studies involving human participants were reviewed and approved by Ethics Committee of Shanghai Pudong New Area Mental Health Center. The patients/participants provided their written informed consent to participate in this study.

## Author Contributions

HG, YH, YJ, CZ, VV, BS, FY and SZ conceived and designed the study. HG, YH and YJ recruited the participants. QD and NH collected the data. QD, NH, JL and HX analyzed the data. JL, HG, HX, CZ, VV, BS, FY and SZ interpreted the data and wrote the paper.

## Funding

This work was supported by the National Natural Science Foundation of China (81771482 to BS); Shanghai Science and Technology Committee (18410710400 to BS); Shanghai Pudong New District Health and Family Planning Commission Key Discipline Construction Fund Project (PWZxk2017-29); Outstanding Clinical Discipline Project of Shanghai Pudong (PWYgy2018-10). Dr. VV is supported by a Medical Research Council Senior Clinical Fellowship (MR/P008747/1). The funding organizations played no further role in study design, data collection, analysis and interpretation and paper writing.

## Conflict of Interest

The authors declare that the research was conducted in the absence of any commercial or financial relationships that could be construed as a potential conflict of interest.

## Abbreviations

DC, degree centrality; EOD, early-onset depression; fMRI, functional magnetic resonance imaging; HAMD, Hamilton Depression Scale; HC, healthy control; LLD, late-life depression; LOD, late-onset depression; MMSE, Mini-Mental State Examination.
