# Supplementary material for: Abnormal Voxel-Wise Degree Centrality in Patients With Late-Life Depression: A Resting-State Functional Magnetic Resonance Imaging Study
Source: Front Psychiatry. 2020 Jan 31;10:1024. doi: 10.3389/fpsyt.2019.01024 (PMC7005207; doi:10.3389/fpsyt.2019.01024)

Table S1. Brain areas showing different DC between LLD patients and HCs (AlphaSim correction of p < 0.05 with a combination of voxel *p* < 0.01 and cluster size > 29).

Region (AAL name)	Peak MNI coordinate			Voxel size	Peak *T* value
	x	*y*	*z*		
**LLD > HC**					
Angular_R / Parietal_Inf_R	42	-57	39	29	4.24
Temporal_Inf_R	63	-30	-21	38	3.87
Temporal_Pole_Sup_R	39	15	-27	36	4.12
Temporal_Pole_Sup_L	-42	9	-18	29	4.06
Parahippocampal Gyrus_R	15	-15	-27	88	5.21
Brainstem_R / L	3	-39	-33	52	4.42
Cerebelum_Crus2_L	-3	-90	-36	94	5.23
**LLD < HC**					
Postcentral_R	18	-33	57	110	-4.60
Postcentral / Precentral_L	-24	-30	54	146	-5.44
Precentral_R	39	-9	42	78	-4.15
Temporal_Mid_R	51	-69	6	31	-4.13
Occipital_Mid_L	-48	-72	3	34	-3.92
Fusiform_L	-24	-81	-15	72	-4.33
Lingual_R	24	-45	-6	34	-4.93
Calcarine_L	-21	-93	18	165	-4.43
Putamen_R	18	21	-15	29	-4.29
Cerebelum_8_R	18	-69	-54	45	-4.28
Cerebelum_8_L	-24	-69	-57	68	-4.22

Table S2. Brain areas showing different DC between LOD patients and EOD patients (AlphaSim correction of p < 0.05 with a combination of voxel *p* < 0.01 and cluster size > 28).

Region (AAL name)	Peak MNI coordinate			Voxel size	Peak *T* value
	x	*y*	*z*		
**LOD > EOD**					
Precuneus_R / L	3	-72	45	50	3.74
Temporal_Mid_R	54	-63	12	85	5.70
ParaHippocampal_R	21	-9	-33	28	3.88
**LOD < EOD**					
Lingual_R	15	-78	-12	58	-4.15
Calcarine_L	-9	-96	-3	31	-3.75
Cuneus_R	12	-93	12	100	-5.41

Figure S1. Brain areas showing difference in DC between LLD patients and HCs (AlphaSim correction of p < 0.05 with a combination of voxel $p < 0.01$ and cluster size > 29), with gender, age, education, score on MMSE and framewise displacement regressed out. Upper: rendering views. Lower: axial slice views. Color bars indicate T-score; a warm color indicates areas where DC value in LLD patients > DC value in HCs, a cold color indicates areas where DC value in LLD patients < DC value in HCs. DC, degree centrality; HC, healthy control; IPL, inferior parietal lobule; ITG, inferior temporal gyrus; L, left; LLD, late-life depression; R, right.

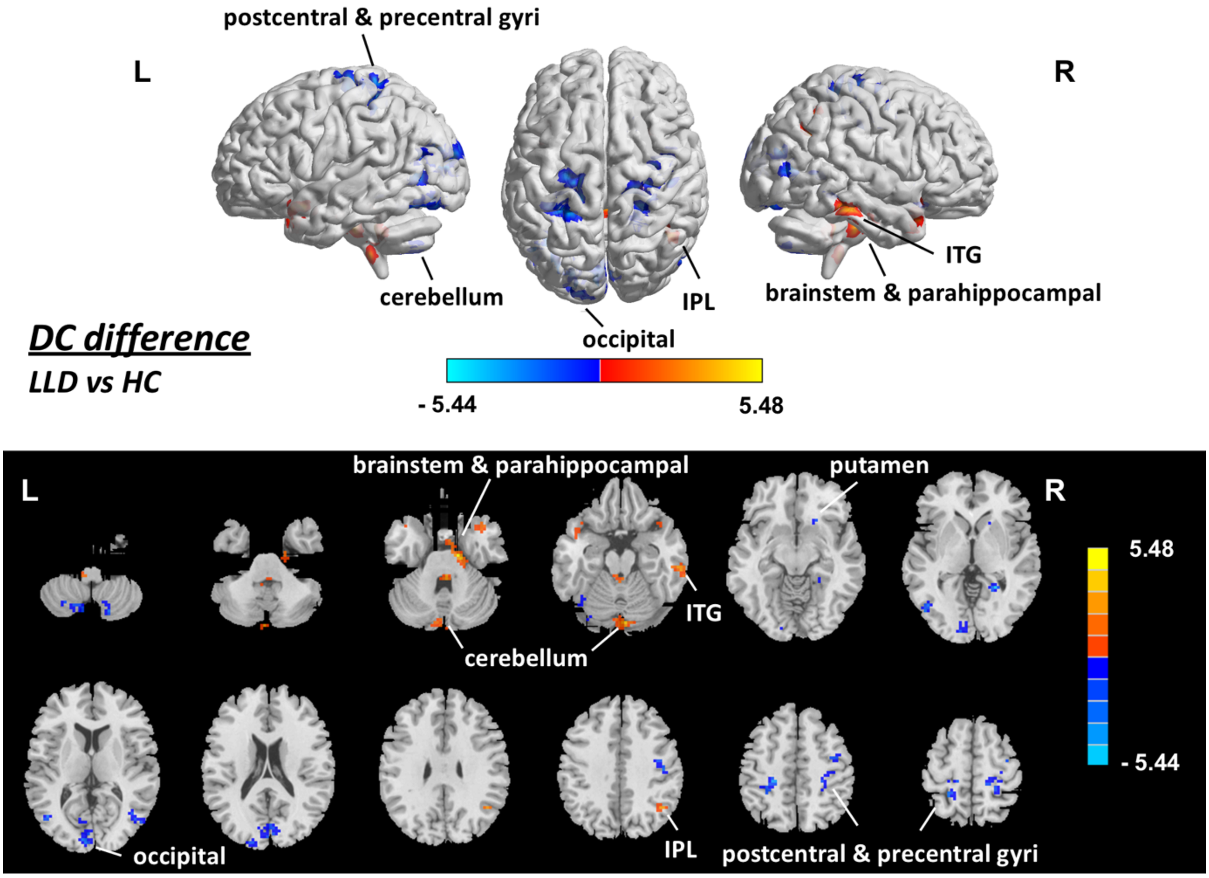

Figure S2. Brain areas showing difference in DC between LOD and EOD patients (AlphaSim correction of p < 0.05 with a combination of voxel *p* < 0.01 and cluster size > 28), with gender, age, education, score on MMSE and framewise displacement regressed out. Upper: rendering views. Lower: axial slice views. Color bars indicate *T*-score; warm color indicates areas whose DC value in LOD patients > DC value in EOD patients, cold color indicates areas whose DC value in LOD patients < DC value in EOD patients. EOD, early-onset depression; HC, healthy control; L, left; LOD, late-onset depression; MTG, middle temporal gyrus; R, right; STG, superior temporal gyrus.

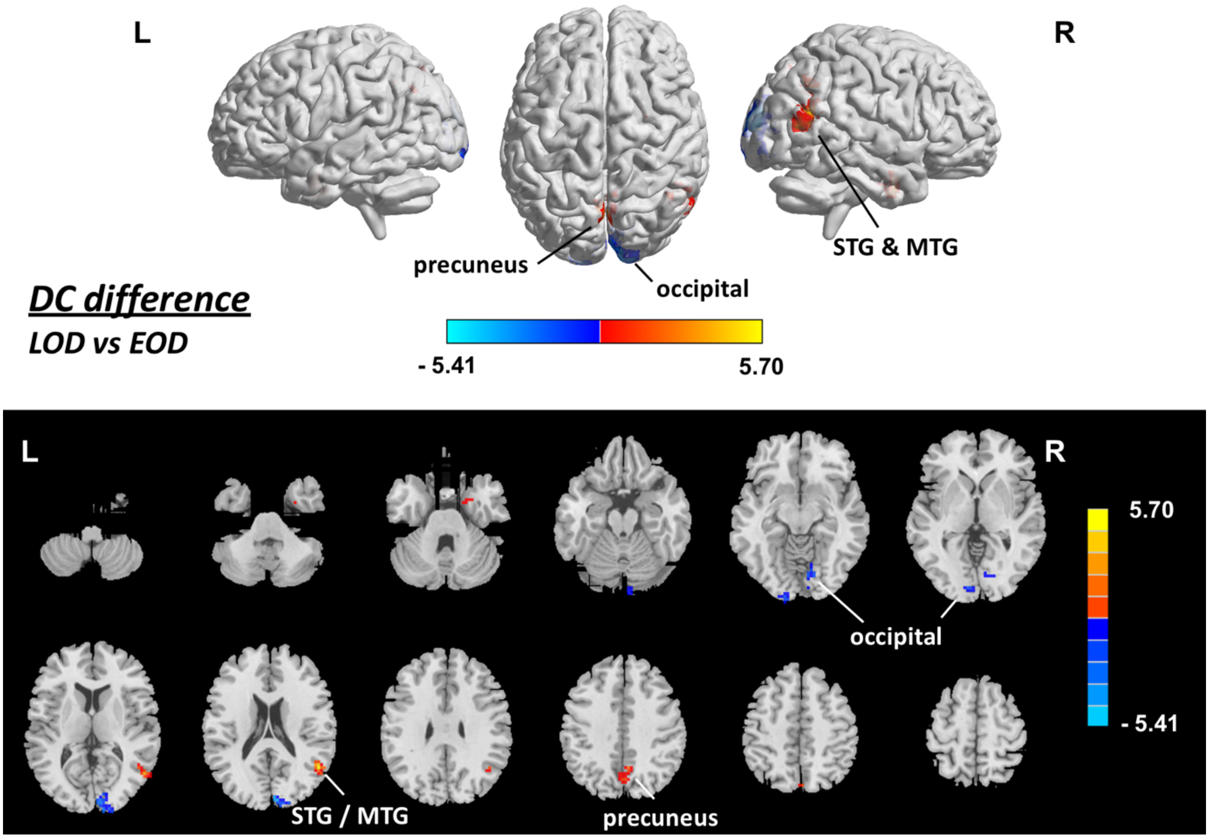

Supplement: Supplementary file 1 [file DataSheet_1.pdf]
